# Trapping of particles diffusing in cylindrical cavity of arbitrary length and radius by two small absorbing disks on the cavity side wall: Narrow escape theory and beyond

**DOI:** 10.1063/5.0211411

**Published:** 2024-07-15

**Authors:** Leonardo Dagdug, Alexander M. Berezhkovskii

**Affiliations:** 1 Departamento de Fisica, Universidad Autonoma Metropolitana-Iztapalapa, 09340 Mexico City, Mexico; 2 Section of Molecular Transport, Eunice Kennedy Shriver National Institute of Child Health and Human Development, National Institutes of Health, Bethesda, Maryland 20819, USA

## Abstract

Narrow escape theory deals with the first passage of a particle diffusing in a cavity with small circular windows on the cavity wall to one of the windows. Assuming that (i) the cavity has no size anisotropy and (ii) all windows are sufficiently far away from each other, the theory provides an analytical expression for the particle mean first-passage time (MFPT) to one of the windows. This expression shows that the MFPT depends on the only global parameter of the cavity, its volume, independent of the cavity shape, and is inversely proportional to the product of the particle diffusivity and the sum of the window radii. Amazing simplicity and universality of this result raises the question of the range of its applicability. To shed some light on this issue, we study the narrow escape problem in a cylindrical cavity of arbitrary size anisotropy with two small windows arbitrarily located on the cavity side wall. We derive an approximate analytical solution for the MFPT, which smoothly goes from the conventional narrow escape solution in an isotropic cavity when the windows are sufficiently far away from each other to a qualitatively different solution in a long cylindrical cavity (the cavity length significantly exceeds its radius). Our solution demonstrates the mutual influence of the windows on the MFPT and shows how it depends on the inter-window distance. A key step in finding the solution is an approximate replacement of the initial three-dimensional problem by an equivalent one-dimensional one, where the particle diffuses along the cavity axis and the small absorbing windows are modeled by delta-function sinks. Brownian dynamics simulations are used to establish the range of applicability of our approximate approach and to learn what it means that the two windows are far away from each other.

## I. INTRODUCTION

The mean lifetime of a particle diffusing in a spherical cavity (more generally, in a cavity without size anisotropy and “pockets” on the cavity wall) with small circular perfectly absorbing disks on the cavity wall is considered by the so-called “narrow escape” (*NE*) theory. Assuming that (1) the disk’s radii are small compared to the local curvature radii of the cavity wall and (2) all the disks are located sufficiently far away from each other and from the particle starting point, the theory provides a universal formula for the mean particle lifetime ${\bar{\tau }}_{NE}$, which is the mean first-passage time to one of the disks,^1^

$${\bar{\tau }}_{NE}=V_{\mathrm{ca}\text{v}}/\left(4D\overset{N}{\underset{i=1}{\sum }}a_{i}\right),$$ (1)

where *V*_*cav*_ is the cavity volume, *D* is the particle diffusivity, *N* is the number of absorbing disks, and *a*_*i*_ are disk’s radii, *i* = 1, 2, …, *N*. To give a simple intuitive derivation of Eq. (1), consider a particle diffusing in such a cavity. Its survival probability *S*(*t*) is the product of the probabilities to avoid being trapped by each of the disks, *S*_*i*_(*t*), $S\left(t\right)=\prod_{i=1}^{N}S_{i}\left(t\right)$. The expression for *S*_*i*_(*t*) is derived in Ref. 2, *S*_*i*_(*t*) = exp[−*k*_*i*_*t*/*V*_*cav*_], where *k*_*i*_ is the Hill–Berg–Purcell^3,4^ rate constant, *k*_*i*_ = 4*Da*_*i*_, which describes the trapping of diffusing particles by a circular absorbing disk of radius *a*_*i*_ located on the otherwise reflecting flat wall. Thus, *S*(*t*) is given by

$$S\left(t\right)=\exp\left[-\overset{N}{\underset{i=1}{\sum }}k_{i}t/V_{\mathrm{ca}\text{v}}\right].$$ (2)

The integral of *S*(*t*) over time from zero to infinity is the mean particle lifetime. Performing the integration, one arrives at the expression in Eq. (1).

According to Eq. (1), ${\bar{\tau }}_{NE}$ depends only on the cavity volume and is independent of its shape and the location of the particle starting point. The simplicity of the above-mentioned formula makes it helpful in analyzing various experimental data and the results of Brownian dynamics simulations of different processes. Examples include transport in spiny dendrites,^5,6^ endosomal sorting,^7^ controlled drug delivery,^8^ channel-mediated membrane transport,^9^ transport in porous media and materials,^10^ and intercellular communication in plants,^11^ to mention just a few. Therefore, this is not surprising that *NE* theory attracted the attention of many researchers. In 2015, Holcman and Schuss published a book on the subject.^12^ One can also find detailed discussion of the *NE* theory in the book by Bressloff.^13^

The goal of the present study was to go beyond the scope of the narrow escape theory. To this end, we study the trapping of a point particle diffusing in a cylindrical cavity with two small absorbing disks located on the cavity side wall, with the focus on the effect of the cavity size anisotropy. A cylindrical cavity has two characteristic sizes: its length and radius. In the absence of the size anisotropy, the mean particle lifetime in such a cavity is given by the narrow escape formula, Eq. (1), unless the disks are not too close to each other. However, as we will see, when the cavity length significantly exceeds its radius, the mean lifetime is the mean particle first-passage time to the cavity cross sections, where the disks are located. A general formula for the mean lifetime in a cavity of arbitrary length and radius is derived, which recovers the above-mentioned results in the corresponding limiting cases. The key step in our derivation is the replacement of the initial three-dimensional problem by an equivalent one-dimensional one. The range of applicability of such a replacement is established by comparing theoretical predictions with the results of Brownian dynamics simulations.

Another issue analyzed in this work is the applicability of the assumption of independent trapping by each of the disks. It is obvious that the assumption fails when the disks are close to one another. The question arises at what distances between the disk centers the assumption provides a reasonable approximation. To answer this question and to learn what it means that two disks are not too close to one another, we also use Brownian dynamics simulations.

The outline of this paper is as follows: a formula for the mean particle lifetime in a cylindrical cavity of arbitrary length and radius with two small absorbing disks on the cavity side wall is derived in Sec. II using the approximate one-dimensional description of particle diffusion in the cavity. Brownian dynamics simulations discussed in Sec. III establish (i) the range of applicability of this one-dimensional description and (ii) at what distance between the disk centers mutual influence of the disks on trapping by each of them can be neglected. Some concluding remarks are made in the final Sec. IV.

## II. THEORY

**FIG. 1.**
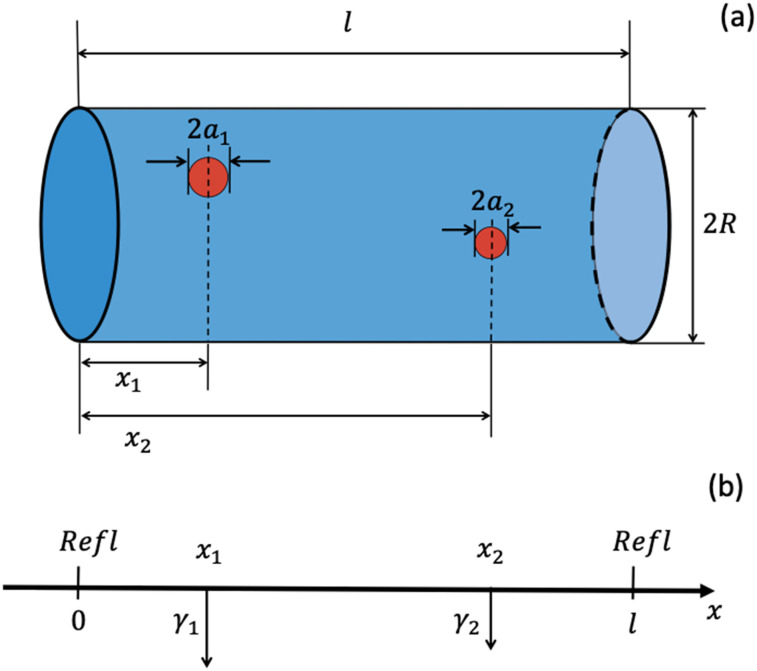
Cylindrical cavity with two perfectly absorbing circular disks on its side wall [panel (a)] and its one-dimensional equivalent [panel (b)].

Consider a point particle diffusing in a cylindrical cavity of length *l* and radius *R* containing two small perfectly absorbing disks of radii *a*_1_ and *a*_2_ on its side wall, *a*_1_, *a*_2_ ≪ *R*. The disk locations are characterized by the *x* coordinates of their centers, *x*_1_ and *x*_2_, with the *x* coordinate measured along the cavity axis; the cavity bases correspond to *x* = 0 and *x* = *l* [see Fig. 1(a)]. The particle is instantly trapped by the disks at their first contact. It is assumed that the particle starting point is uniformly distributed over the cavity volume. Let *p*(**r**, *t*) be the probability density of finding the particle at point **r** at time *t*; *p*(**r**, 0) = 1/*V*_*cav*_. This probability density satisfies the diffusion equation,

$$\frac{\partial p}{\partial t}=D\nabla^{2}p,$$ (3)

with reflecting boundary conditions on the cavity walls and absorbing boundary conditions on the discs. The particle survival probability *S*(*t*) is the integral of *p*(**r**, *t*) over the cavity volume,

$$S\left(t\right)=\int_{V_{\mathrm{ca}\text{v}}}p\left(r,t\right)dr.$$ (4)

The particle lifetime probability density *φ*(*t*) and its mean lifetime $\bar{\tau }$ are given by

φ(t)=−dS(t)/dt (5)

and

$$\bar{\tau }=\int_{0}^{\infty }t\varphi \left(t\right)dt=\int_{0}^{\infty }S\left(t\right)dt.$$ (6)

Thus, to find $\bar{\tau }$, one has to solve Eq. (3) with the initial and boundary conditions mentioned above.

An exact solution to this problem and hence for the mean lifetime are unknown. Therefore, we find an approximate solution for $\bar{\tau }$ using the facts that (i) particle motion in the cavity without absorbing disks may be considered as one-dimensional diffusion along the cavity axis and (ii) the small disks are localized perturbations. Specifically, we model the absorbing disks as delta-function sinks [see Fig. 1(b)] with sink intensities *γ*_1_ and *γ*_2_, which are functions of the disk radii given by^14^

$$\gamma_{1}=\frac{4Da_{1}}{\pi R^{2}},\;\gamma_{2}=\frac{4Da_{2}}{\pi R^{2}}.$$ (7)

Earlier, we applied such a one-dimensional approach to treat particle diffusion in a cylindrical cavity with one small absorbing disk on the cavity side wall.^15^ Here, we extend the approach to the case of two small absorbing disks.

To be trapped, the particle first has to reach the cross sections *x* = *x*_1_ or *x* = *x*_2_, where the disks are located. Let ${\bar{\tau }}_{FP}$ and ${\bar{\tau }}_{tr}$ be the mean first-passage time to these cross sections and the mean trapping time of trajectories starting from these cross sections, respectively. The mean particle lifetime $\bar{\tau }$ is the sum of ${\bar{\tau }}_{FP}$ and ${\bar{\tau }}_{tr}$,

$$\bar{\tau }={\bar{\tau }}_{FP}+{\bar{\tau }}_{tr}.$$ (8)

The mean first-passage time ${\bar{\tau }}_{FP}$ is a weighted sum of three terms: ${\bar{\tau }}_{FP}\left(\left.x_{0}\to x_{1}\right|x_{0}<x_{1}\right)$, ${\bar{\tau }}_{FP}\left(x_{1}\leftarrow x_{0}\to x_{2}\right)$, and ${\bar{\tau }}_{FP}\left(\left.x_{0}\to x_{2}\right|x_{0}>x_{2}\right)$, where *x*_0_ is the particle staring point; ${\bar{\tau }}_{FP}\left(\left.x_{0}\to x_{1}\right|x_{0}<x_{1}\right)$ is the mean first-passage time to *x*_1_ for trajectories starting on the left of *x*_1_, 0 < *x*_0_ < *x*_1_; ${\bar{\tau }}_{FP}\left(x_{1}\leftarrow x_{0}\to x_{2}\right)$ is the mean first-passage time to *x*_1_ or *x*_2_ for trajectories starting in between *x*_1_ and *x*_2_, *x*_1_ < *x*_0_ < *x*_2_; ${\bar{\tau }}_{FP}\left(\left.x_{0}\to x_{2}\right|x_{0}>x_{2}\right)$ is the mean first-passage time to *x*_2_ for trajectories starting on the right of *x*_2_, *x*_2_ < *x*_0_ < *l*; and the bar above *τ*_*FP*_ indicates averaging over *x*_0_. These mean first-passage times are given by (see the Appendix)

$${\bar{\tau }}_{FP}\left(\left.x_{0}\to x_{1}\right|x_{0}<x_{1}\right)=\frac{x_{1}^{2}}{3D},$$ (9)

$${\bar{\tau }}_{FP}\left(x_{1}\leftarrow x_{0}\to x_{2}\right)=\frac{{\left(x_{2}-x_{1}\right)}^{2}}{12D},$$ (10)

and

$${\bar{\tau }}_{FP}\left(\left.{\;x}_{0}\to x_{2}\right|x_{0}>x_{2}\right)=\frac{{\left(l-x_{2}\right)}^{2}}{3D}.$$ (11)

Because of the uniform initial distribution of the particle starting point over the cavity volume, the corresponding weight factors are *x*_1_/*l*, $\left(x_{2}-x_{1}\right)/l$, and $\left(l-x_{2}\right)/l$, respectively. Thus, we have

$$\begin{array}{ll} {\bar{\tau }}_{FP} & =\frac{1}{l}\left[x_{1}{\bar{\tau }}_{FP}\left(\left.{\;x}_{0}\to x_{1}\right|x_{0}\left\langle x_{1}\;\right.\right)+\left(x_{2}-x_{1}\right){\bar{\tau }}_{FP}\left(x_{1}\leftarrow x_{0}\to x_{2}\right)\right.\\ & \;\left.+\left(l-x_{2}\right){\bar{\tau }}_{FP}\left(\left.{\;x}_{0}\to x_{2}\right|x_{0}>x_{2}\right)\right]. \end{array}$$ (12)

Substituting here the expressions for the mean first-passage times, Eqs. (9)–(11), after some manipulations we arrive at

$$\begin{array}{ll} {\bar{\tau }}_{FP} & =\frac{1}{12lD}\left[4x_{1}^{3}+{\left(x_{2}-x_{1}\right)}^{3}+4{\left(l-x_{2}\right)}^{3}\right]\\ & =\frac{l^{2}}{12D}\left[4\nu_{1}^{3}+{\left(\nu_{2}-\nu_{1}\right)}^{3}+4{\left(1-\nu_{2}\right)}^{3}\right], \end{array}$$ (13)

where *ν*_1,2_ = *x*_1,2_/*l*.

The mean trapping time ${\bar{\tau }}_{tr}$ is also a weighted sum of the mean trapping times *τ*_*tr*_(*x*_1_) and *τ*_*tr*_(*x*_2_) of trajectories starting from the cross sections *x* = *x*_1_ and *x* = *x*_2_, respectively. The corresponding weight factors *W*_1_ and *W*_2_ in this case are

$$\begin{array}{c} W_{1}=\frac{1}{l}\left[x_{1}+\left(x_{2}-x_{1}\right)/2\right]=\frac{x_{1}+x_{2}}{2l},\\ W_{2}=\frac{1}{l}\left[l-x_{2}+\left(x_{2}-x_{1}\right)/2\right]=\frac{2l-x_{1}-x_{2}}{2l}. \end{array}$$ (14)

Thus, we have

$${\bar{\tau }}_{tr}=\frac{1}{2l}\left[\left(x_{1}+x_{2}\right)\tau_{tr}\left(x_{1}\right)+\left(2l-x_{1}-x_{2}\right)\tau_{tr}\left(x_{2}\right)\right].$$ (15)

**FIG. 2.**
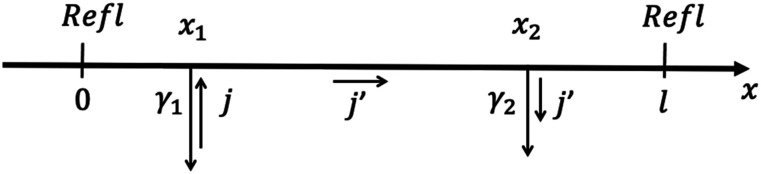
Steady state used to derive the mean trapping time *τ*_*tr*_(*x*_1_).

To derive an expression for *τ*_*tr*_(*x*_1_), consider a steady state maintained by a constant flux *j* injected at point *x*_1_, as shown in Fig. 2. The mean trapping time *τ*_*tr*_(*x*_1_) is equal to the ratio of the steady-state number of particles in the cavity *N* to this flux,^16^

$$\tau_{tr}\left(x_{1}\right)=\frac{1}{j}N,\;N=\int_{0}^{l}c\left(x\right)dx,$$ (16)

where *c*(*x*) is the steady-state concentration of the particles. This concentration is constant for 0 < *x* < *x*_1_ and *x*_2_ < *x* < *l*, where it is equal to *c*(*x*_1_) and *c*(*x*_2_), respectively. In the interval *x*_1_ < *x* < *x*_2_, the concentration satisfies

$$-\;D\frac{dc\left(x\right)}{dx}=j^{\prime },$$ (17)

where *j*′ is the steady-state flux in this interval, given by

$$j^{\prime }=j-\gamma_{1}c\left(x_{1}\right)=\gamma_{2}c\left(x_{2}\right).$$ (18)

Solving Eqs. (17) and (18), we obtain

$$j^{\prime }=\frac{\gamma_{2}}{\gamma_{1}+\gamma_{2}+\gamma_{1}\gamma_{2}\left(x_{2}-x_{1}\right)/D}\;j$$ (19)

and

$$c\left(x\right)=j^{\prime }\times \left\{\begin{array}{c} 1/\gamma_{2}+\left(x_{2}-x_{1}\right)/D,\;0<x<x_{1}\;\\ 1/\gamma_{2}+\left(x_{2}-x\right)/D,\;x_{1}<x<x_{2}\;\\ 1/\gamma_{2},\;x_{2}<x<l\;.\; \end{array}\right.$$ (20)

Substituting *c*(*x*) in Eq. (20) into the definition of *N* in Eq. (16), performing the integration, and using the relationship between *j* and *j*′ in Eq. (19), we arrive at

$$\tau_{tr}\left(x_{1}\right)=\frac{l+\gamma_{2}\left(x_{2}^{2}-x_{1}^{2}\right)/\left(2D\right)}{\gamma_{1}+\gamma_{2}+\gamma_{1}\gamma_{2}\left(x_{2}-x_{1}\right)/D}.$$ (21)

We can write an expression for *τ*_*tr*_(*x*_2_) by analogy with Eq. (21) using the observation that $x_{2}^{2}-x_{1}^{2}=\left(x_{2}-x_{1}\right)\left(x_{1}+x_{2}\right)$, where *x*_2_ − *x*_1_ is the distance between the disk centers and *x*_1_ + *x*_2_ is the sum of the distances from the sinks to the left cavity base. Keeping this in mind, we replace the second term in the numerator of Eq. (21) by $\gamma_{1}\left(x_{2}-x_{1}\right)\left(2l-x_{1}-x_{2}\right)/\left(2D\right)$, where 2*l* − *x*_1_ − *x*_2_ is the sum of the distances from the sinks to the right base of the cavity. The result is

$$\tau_{tr}\left(x_{2}\right)=\frac{l+\gamma_{1}\left(x_{2}-x_{1}\right)\left(2l-x_{1}-x_{2}\right)/\left(2D\right)}{\gamma_{1}+\gamma_{2}+\gamma_{1}\gamma_{2}\left(x_{2}-x_{1}\right)/D}.$$ (22)

Finally, replacing in Eqs. (21) and (22), *γ*_1_ and *γ*_2_ by their expressions in terms of the disks and cavity radii in Eq. (7), and substituting the results into Eq. (15), we arrive at

$${\bar{\tau }}_{tr}=\frac{V_{\mathrm{ca}\text{v}}+\frac{x_{2}-x_{1}}{l}\left[a_{1}{\left(2l-x_{1}-x_{2}\right)}^{2}+a_{2}{\left(x_{1}+x_{2}\right)}^{2}\right]}{4D\left[a_{1}+a_{2}+\frac{4a_{1}a_{2}}{\pi R^{2}}\left(x_{2}-x_{1}\right)\right]},$$ (23)

where *V*_*cav*_ = *πR*^2^*l* is the cavity volume. When the disk radii tend to zero, *a*_1_, *a*_2_ → 0, ${\bar{\tau }}_{tr}$ reduces to the mean narrow escape time, Eq. (1), which, in our case, is given by

$${\left.{\bar{\tau }}_{tr}\right|}_{a_{1},a_{2}\to 0}={\bar{\tau }}_{NE}=\frac{V_{\mathrm{ca}\text{v}}}{4D\left(a_{1}+a_{2}\right)}=\frac{\pi R^{2}l}{4D\left(a_{1}+a_{2}\right)}.$$ (24)

We use ${\bar{\tau }}_{NE}$ in Eq. (24) to write ${\bar{\tau }}_{tr}$ in Eq. (23), as

$${\bar{\tau }}_{tr}={\bar{\tau }}_{NE}\frac{1+\frac{x_{2}-x_{1}}{\pi R^{2}l^{2}}\left[a_{1}{\left(2l-x_{1}-x_{2}\right)}^{2}+a_{2}{\left(x_{1}+x_{2}\right)}^{2}\right]}{1+\frac{x_{2}-x_{1}}{\pi R^{2}}\frac{4a_{1}a_{2}}{a_{1}+a_{2}}}.$$ (25)

From this, one can see that when the disks radii are equal, *a*_1_ = *a*_2_, the mean trapping time considered as a function of *x*_1_ and *x*_2_ has a minimum when these distances satisfy *x*_1_ = *l* − *x*_2_, and its minimum value is ${\bar{\tau }}_{NE}$.

Expressions in Eqs. (8), (13), (24), and (25) give the mean particle lifetime $\bar{\tau }$ in terms of the cavity parameters *l* and *R*, the disk radii *a*_1_ and *a*_2_, and the distances *x*_1_ and *x*_2_ of the disk centers from the left base of the cavity. This is one of the main results of the present work. As follows from Eqs. (24) and (25), the mean trapping time is proportional to the cavity length *l*, whereas the mean first-passage time, Eq. (13), is proportional to *l*^2^. The latter provides the asymptotic (*l* → *∞*) behavior of the mean particle lifetime in a cylindrical cavity with two small absorbing disks on the cavity side wall, Eq. (8), which is independent of the disk’s radii. Thus, we have

$$\bar{\tau }\approx \left\{\begin{array}{c} {\bar{\tau }}_{NE}=\frac{\pi R^{2}l}{4D\left(a_{1}+a_{2}\right)},\;l<\frac{3\pi R^{2}}{a_{1}+a_{2}},\;\\ {\bar{\tau }}_{FP}=\frac{l^{2}}{12D}\left[4\nu_{1}^{3}+{\left(\nu_{2}-\nu_{1}\right)}^{3}+4{\left(1-\nu_{2}\right)}^{3}\right],\;l\to \infty .\; \end{array}\right.$$ (26)

**FIG. 3.**
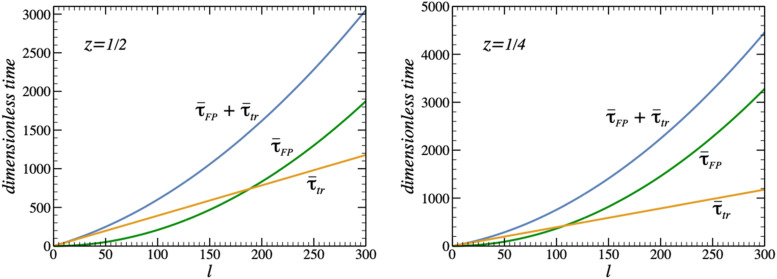
Dependences of ${\bar{\tau }}_{FP}$, ${\bar{\tau }}_{tr}$, and their sum $\bar{\tau }$ measured in units *R*^2^/*D* on the cavity length *l* measured in units of *R* for *a*_1_ = *a*_2_ = 0.1*R* and $x_{1,2}=l\left(1\mp z\right)/2$ at *z* = 1/4 and 1/2.

It is worth noting that the transition between the two asymptotic behaviors with the increasing cavity length *l* occurs very slowly. To illustrate this in Fig. 3, we show the two terms ${\bar{\tau }}_{FP}$ and ${\bar{\tau }}_{tr}$ contributing to the mean lifetime $\bar{\tau }$, Eq. (8). The *l*-dependences are shown in dimensionless units, i.e., we take *D* = *R* = 1 and measure the cavity length *l* in units of *R* and time in units of *R*^2^/*D*. For illustrative purposes, we choose *a*_1_ = *a*_2_ = 0.1*R* and $x_{1,2}=l\left(1\mp z\right)/2$, where $z=\left(x_{2}-x_{1}\right)/l$ and *z* = 1/2 and 1/4. For this choice of parameters, Eqs. (13) and (25) take the form

$${\bar{\tau }}_{FP}=\left[{\left(z-1/2\right)}^{2}+1/12\right]\frac{l^{2}}{4}$$ (13a)

and

$${\bar{\tau }}_{tr}={\bar{\tau }}_{NE}=\frac{5\pi }{4}l.$$ (25a)

In Fig. 3, we also show their sum $\bar{\tau }={\bar{\tau }}_{FP}+{\bar{\tau }}_{tr}$. As follows from Eq. (13a), ${\bar{\tau }}_{FP}$ considered as a function of *z* has a minimum at *z* = 1/2, i.e., when *x*_1_ = *l*/4 and *x*_2_ = 3*l*/4. Since for the disks of equal radii located at equal distances from the cavity bases, *x*_1_ = *l* − *x*_2_, ${\bar{\tau }}_{tr}$ is independent of *x*_1_ and *x*_2_ and equal to ${\bar{\tau }}_{NE}$, the mean particle lifetime $\bar{\tau }$ also has a minimum at *x*_1_ = *l*/4, *x*_2_ = 3*l*/4, and its minimum value is ${\bar{\tau }}_{\min}=\left(l^{2}+60\pi l\right)/48$.

## III. SIMULATIONS

The above-mentioned results are obtained using the approximate one-dimensional description of the trapping problem. To establish the range of its applicability, we run Brownian dynamics simulations. The aim of our simulations is twofold: (1) to establish the range of the disk radius, where the replacement of a small absorbing disk on the cavity wall by a delta-function sink with the sink intensity given by Eq. (7) is justifiable and (2) to study at what inter-disk distances our approximate treatment of trapping by two disks is applicable.

To clarify the first issue, we consider the case of the only absorbing disk of radius *a* on the side wall of the cylindrical cavity of length *l* and radius *R*. The *x* coordinate of the disk center is denoted by *x*_*a*_. In this case, our theory with *a*_1_ = *a*, *a*_2_ = 0, and *x*_1_ = *x*_*a*_ leads to

$$\bar{\tau }=\frac{V_{\mathrm{ca}\text{v}}}{4Da}+\frac{l^{2}-3lx_{a}+3x_{a}^{2}}{3D}=\frac{1}{12D}\left[3\pi \frac{R^{2}l}{a}+4\left(l^{2}-3lx_{a}+3x_{a}^{2}\right)\right].$$ (27)

We compare the predictions of this formula to the simulation results to establish constraints on (1) the disk radius *a*, (2) the cavity length *l*, and (3) the *x* coordinate of the disk center *x*_*a*_. For comparison, it is convenient to use the cavity radius *R* as a length scale and to measure the mean particle lifetime in units of $R^{2}/\left(12D\right)$. The theory predicts that this time is given by [see Eq. (27)]

$${\bar{\tau }}_{th}=\frac{12D\bar{\tau }}{R^{2}}=3\pi \frac{l}{a}+4\left[{\left(\frac{l}{R}\right)}^{2}-3\frac{l}{R}\frac{x_{a}}{R}+3{\left(\frac{x_{a}}{R}\right)}^{2}\right].$$ (28)

We start our comparison of the theoretical predictions and simulation results with the case of *x*_*a*_ = *l*/2, in cavities of lengths *l*/*R* = 0.5, 1, 2, and 4, with the disks of radii *a*/*R* = 0.2, 0.15, 0.1, 0.05, and 0.01. The ratios of the theoretical predictions and simulation results are presented in Table I. One can see that the replacement of a small absorbing disk by the delta-function sink is applicable when *a* ≤ 0.1*R* and *l* ≥ 2*R*. Note that this is true under the condition that *x*_*a*_ = *l*/2.

**TABLE I.** Ratio of the mean particle lifetime in a cylindrical cavity of length *l* and radius *R* with an absorbing disk of radius *a* on the cavity side wall equally spaced from both cavity bases predicted by the theory to its counterpart obtained in three-dimensional Brownian dynamics simulations as a function of the disk radius for cavities with *l*/*R* = 0, 5, 1, 2, and 4.

*a*/*R*	*l*/*R*			
	0.5	1	2	4
0.20	0.716	1.067	1.077	1.098
0.15	0.769	1.048	1.050	1.051
0.10	0.862	1.022	1.017	0.992
0.05	0.878	0.967	1.001	1.001
0.01	0.853	0.981	0.995	0.997

Next, we use simulations to check the *x*_*a*_-dependence of the mean lifetime $\bar{\tau }$ predicted by Eq. (27) and its dimensionless version, Eq. (28). This is done for the absorbing disk of radius *a* = 0.05*R* for two cavities of length *l* = 2*R* and *l* = 4*R*. In both cases, we compare the theoretical predictions and simulation results for *x*_*a*_ = 0.1*R*, 0.2*R*, 0.3*R*, and 0.5*R*. The ratios of the theoretical predictions and simulation results are presented in Table II. One can see that the replacement of the small absorbing disk by the delta-function sink is applicable when *x*_*a*_ equals or exceeds 0.3*R*, *x*_*a*_ ≥ 0.3*R*.

**TABLE II.** Ratio of the mean particle lifetime in a cylindrical cavity of length *l* and radius *R* with an absorbing disk of radius *a* = 0.05*R* located on the cavity side wall at distance *x*_*a*_ from the left cavity base predicted by the theory to its counterpart obtained in three-dimensional Brownian dynamics simulations as a function of *x*_*a*_ for cavities with *l*/*R* = 2 and 4.

*x*_*a*_/*R*	*l*/*R*	
	2	4
0.1	0.884	0.889
0.2	0.949	0.950
0.3	0.973	0.975
0.5	0.992	0.993

Our approximate treatment of trapping by two disks obviously fails when the disks are close to each other since it does not recover the mean lifetime in the presence of a single disk when the disk centers coincide. Nevertheless, it provides a good approximation for $\bar{\tau }$ when the disks are not too close. Since the disks are small, *a*_1_, *a*_2_ ≪ *R*, this is possible even when the *x* coordinates of their centers are equal, *x*_1_ = *x*_2_, on the condition that they are located at the opposite ends of the diameter of the cavity cross section normal to its axis.

To clarify this issue, we take advantage of Brownian dynamics simulations to study the trapping of a point particle diffusing in a cylindrical cavity of length *l* and radius *R* with two small absorbing disks of the same radius *a*_1_ = *a*_2_ = *a* = 0.05*R* on the side cavity wall. The disk centers are equally distant from both cavity bases, *x*_1_ = *x*_2_ = *l*/2. The cavity cross section passing through the disk centers is a circle of radius *R*. Let **r**_1_ and **r**_2_ be vectors connecting the center of this circle with the disk centers and *φ* be the angle between **r**_1_ and **r**_2_. The disks coincide when *φ* = 0 and are on the opposite ends of the circle diameter when *φ* = *π*.

When *x*_1_ = *x*_2_ = *l*/2, Eqs. (13) and (23) reduce to

$${\left.{\bar{\tau }}_{FP}\right|}_{x_{1}=x_{2}=l/2}=l^{2}/\left(12D\right),\;{\bar{\tau }}_{tr}=V_{\mathrm{ca}\text{v}}/\left(8Da\right),$$ (29)

and our one-dimensional theory predicts

$$\bar{\tau }=\frac{V_{\mathrm{ca}\text{v}}}{8Da}+\frac{l^{2}}{12D}=\frac{\pi R^{2}l}{8Da}+\frac{l^{2}}{12D}.$$ (30)

We compare this theoretical prediction to the simulation results obtained for two cavities of length *l* = 2*R* and *l* = 4*R* at eight values of angle *φ*: *φ* = *π*, 3*π*/4, *π*/2, *π*/4, *π*/8, *π*/16, *π*/32, and *π*/64. For comparison, we use the dimensionless form of the mean particle lifetime [see Eq. (28)]

$${\bar{\tau }}_{th}=\frac{12D\bar{\tau }}{R^{2}}=\frac{3\pi l}{2a}+{\left(\frac{l}{R}\right)}^{2}.$$ (31)

The ratios of the theoretical predictions and simulation results are presented in Table III. One can see that mutual influence of the disks on trapping by each of them can be neglected when *φ* > *π*/16, and the distance between the disk centers exceed five disk radii.

**TABLE III.** Ratio of the mean particle lifetime in a cylindrical cavity of length *l* and radius *R* with two absorbing disk of radius *a* = 0.05*R* located on the cavity side wall equally spaced from the cavity bases predicted by the theory to its counterpart obtained in three-dimensional Brownian dynamics simulations as a function of angle *φ* (see the text) for cavities with *l*/*R* = 2 and 4.

*φ*	*l*/*R*	
	2	4
*π*	1.008	1.001
3*π*/4	0.996	1.003
*π*/2	1.004	0.992
*π*/4	1.005	1.007
*π*/8	1.009	1.008
*π*/16	0.979	0.973
*π*/32	0.715	0.707
*π*/64	0.601	0.593

## IV. CONCLUDING REMARKS

This work deals with the trapping of a point particle diffusing in a cylindrical cavity by two small perfectly absorbing circular disks of different radii located on the cavity side wall. Assuming that the particle starting point is uniformly distributed over the cavity volume, we derive an approximate expression for the mean particle lifetime, which is its mean first-passage time (MFPT), to one of the disks. The expression gives the MFPT as a function of the cavity length *l* and radius *R*, radii of the disks *a*_1_ and *a*_2_, and the distances *x*_1_ and *x*_2_ of the disk centers from the left cavity base. It shows that the narrow escape theory provides a good estimate for the MFPT, Eq. (24), in cavities with no size anisotropy and fails in highly anisotropic cavities, as might be expected. As follows from Eq. (24), in the absence of the size anisotropy, the MFPT scales as the cavity volume *V*_*cav*_ = *πR*^2^*l*, i.e., it is proportional to *l* and *R*^2^, while in highly anisotropic cavities, it scales as *l*^2^, Eq. (13), independent of the cavity radius. In these limiting cases, corrections to the corresponding asymptotic behaviors of the MFPT are small and can be neglected.

Our analysis is based on the approximate replacement of the initial three-dimensional problem by an equivalent one-dimensional one, where the particle diffuses along the cavity axis and the small absorbing disks are modeled as delta-function sinks with the sink intensities given in Eq. (7). To establish the range of applicability of this replacement, we run three-dimensional Brownian dynamics simulations and compare the simulation results with the theoretical predictions made in the framework of the one-dimensional theory. The comparison shows that the replacement is justifiable when (i) the disk radii do not exceed 0.1*R* and (ii) the distances from the disk centers to the civility bases exceed three disk radii.

As might be expected, the disks noticeably affect trapping by each other when they are close. In the simulations, we found that this effect can be neglected when the distance between the disk centers exceeds five disk radii.

When the above-mentioned conditions are satisfied, our theory provides an expression for the mean lifetime at an arbitrary level of the cavity size anisotropy. Finally, we note that our approach can be straightforwardly generalized to the case of more than two small absorbing disks on the cavity side wall.

## Data Availability

The data that support this study are available from the corresponding author upon a reasonable request.

## APPENDIX: MEAN FIRST-PASSAGE TIMES ${\bar{\tau }}_{FP}\left(\left.x_{0}\to x_{1}\right|x_{0}<x_{1}\right)$, ${\bar{\tau }}_{FP}\left(x_{1}\leftarrow x_{0}\to x_{2}\right)$, AND ${\bar{\tau }}_{FP}\left(\left.x_{0}\to x_{2}\right|x_{0}>x_{2}\right)$

The mean lifetime $\tau_{FP}\left(a\leftarrow x_{0}\to b\right)$ of a particle diffusing in the interval *a* ≤ *x* ≤ *b* terminated by two absorbing points is given by

$$\tau_{FP}\left(a\leftarrow x_{0}\to b\right)=\frac{\left(x_{0}-a\right)\left(b-x_{0}\right)}{2D},$$ (A1)

where *x*_0_ is the particle starting point, *a* < *x*_0_ < *b*. This can be obtained using the fact that $\tau_{FP}\left(a\leftarrow x_{0}\to b\right)$ satisfies the equation,

$$\frac{d^{2}\tau_{FP}\left(a\leftarrow x_{0}\to b\right)}{d{x_{0}}^{2}}=-1$$ (A2)

and absorbing boundary conditions at the end points,

$${\left.\tau_{FP}\left(a\leftarrow x_{0}\to b\right)\right|}_{x_{0}=a}={\left.\tau_{FP}\left(a\leftarrow x_{0}\to b\right)\right|}_{x_{0}=b}=0.$$ (A3)

The mean lifetime ${\bar{\tau }}_{FP}\left(a\leftarrow x_{0}\to b\right)$ is the mean lifetime in Eq. (A1) averaged over *x*_0_,

$${\bar{\tau }}_{FP}\left(a\leftarrow x_{0}\to b\right)=\frac{1}{b-a}\int_{a}^{b}\tau_{FP}\left(a\leftarrow x_{0}\to b\right)dx_{0}.$$ (A4)

Performing the integration, we arrive at

$${\bar{\tau }}_{FP}\left(a\leftarrow x_{0}\to b\right)=\frac{{\left(b-a\right)}^{2}}{12D}.$$ (A5)

When one of the end points of the interval is reflecting, say point *b*, we deal with the mean first-passage time $\tau_{FP}^{\left(ref\right)}\left(x_{0}\to a\right)$, which also satisfies Eq. (A2) with the absorbing boundary condition at point *a* and the reflecting boundary condition at point *b*,

$${\left.\frac{d\tau_{FP}^{\left(ref\right)}\left(x_{0}\to a\right)}{dx_{0}}\right|}_{x_{0}=b}=0.$$ (A6)

Solving Eq. (A2) with such boundary conditions, one finds

$$\tau_{FP}^{\left(ref\right)}\left(x_{0}\to a\right)=\frac{\left(x_{0}-a\right)\left(2b-a-x_{0}\right)}{2D}.$$ (A7)

When point *a* is reflecting, we deal with the mean first-passage time $\tau_{FP}^{\left(ref\right)}\left(x_{0}\to b\right)$, which is given by

$$\tau_{FP}^{\left(ref\right)}\left(x_{0}\to b\right)=\frac{\left(b-x_{0}\right)\left(x_{0}-2a+b\right)}{2D}.$$ (A8)

We find the mean lifetimes ${\bar{\tau }}_{FP}^{\left(ref\right)}\left(x_{0}\to a\right)$ and ${\bar{\tau }}_{FP}^{\left(ref\right)}\left(x_{0}\to b\right)$ by averaging $\tau_{FP}^{\left(ref\right)}\left(x_{0}\to a\right)$ and $\tau_{FP}^{\left(ref\right)}\left(x_{0}\to b\right)$ over *x*_0_. This leads to

$${\bar{\tau }}_{FP}^{\left(ref\right)}\left(x_{0}\to b\right)=\frac{1}{b-a}\int_{a}^{b}\tau_{FP}^{\left(ref\right)}\left(x_{0}\to b\right)dx_{0}=\frac{{\left(b-a\right)}^{2}}{3D}$$ (A9)

and

$${\bar{\tau }}_{FP}^{\left(ref\right)}\left(x_{0}\to a\right)=\frac{1}{b-a}\int_{a}^{b}\tau_{FP}^{\left(ref\right)}\left(x_{0}\to a\right)dx_{0}=\frac{{\left(b-a\right)}^{2}}{3D}.$$ (A10)

Using the results in Eqs. (A5), (A9), and (A10), one can find the mean first-passage times of interest, ${\bar{\tau }}_{FP}\left(\left.x_{0}\to x_{1}\right|x_{0}<x_{1}\right)$, ${\bar{\tau }}_{FP}\left(x_{1}\leftarrow x_{0}\to x_{2}\right)$, and ${\bar{\tau }}_{FP}\left(\left.x_{0}\to x_{2}\right|x_{0}>x_{2}\right)$, given in Eqs. (9)–(11).
